# Rapid Corneal Endothelial Cell Loss After PreserFlo MicroShunt Surgery: A Case Report

**DOI:** 10.7759/cureus.83091

**Published:** 2025-04-27

**Authors:** Kentaro Iwasaki, Yoshihiro Takamura, Masaru Inatani

**Affiliations:** 1 Department of Ophthalmology, Faculty of Medical Sciences, University of Fukui, Fukui, JPN

**Keywords:** corneal endothelial cell, glaucoma surgery, intraocular pressure, preserflo microshunt, trabeculectomy

## Abstract

We describe a rare case of rapid endothelial cell loss after PreserFlo MicroShunt (PMS) surgery, necessitating tube removal and conversion to trabeculectomy. An 85-year-old female patient with advanced bilateral primary open-angle glaucoma underwent PMS surgery in the left eye. The surgery was uneventful, and the initial postoperative course was stable, with intraocular pressure (IOP) maintained between 6 mmHg and 14 mmHg without glaucoma medication. However, despite stable IOP control, corneal endothelial cell density (CECD) declined rapidly postoperatively, decreasing from 2075 cells/mm² preoperatively to 1069 cells/mm² within 11 months. Given the progressive endothelial cell loss, PMS removal and conversion to trabeculectomy were performed in the same quadrant. Following trabeculectomy, the patient initially experienced excessive filtration, which led to hypotensive maculopathy and severe choroidal detachment. This was successfully managed with transconjunctival sutures. After stabilization, IOP remained at 8 mmHg six months postoperatively. Importantly, the rapid decline in CECD ceased following PMS removal, with CECD measuring 1038 cells/mm² six months after trabeculectomy. This case highlights the potential risk of rapid endothelial cell loss after PMS surgery and emphasizes the importance of close monitoring of endothelial cells. PMS removal may be necessary in cases of progressive CECD loss to prevent further corneal decompensation.

## Introduction

The PreserFlo MicroShunt (PMS) (Santen Pharmaceutical Co. Ltd., Osaka, Japan) is a microinvasive filtration surgical device approved for use in Japan since February 2022. It is an aqueous humor drainage shunt designed to be implanted using an ab externo approach (from outside the eye), creating a full-thickness fistula (drainage tunnel) from the anterior chamber to the subconjunctival space and forming a drainage reservoir called a filtering bleb to lower intraocular pressure (IOP). PMS surgery is considered a less invasive filtration procedure than trabeculectomy, as it does not require scleral flap creation or iridectomy [1]. Notably, PMS surgery has been associated with fewer postoperative interventions and a lower incidence of hypotony than trabeculectomy; however, its IOP-lowering effect is less pronounced [2]. As a result, PMS surgery is increasingly being performed as an alternative to trabeculectomy for patients with glaucoma and medically uncontrollable IOP [3].

Previous reports have evaluated the corneal endothelial cell loss following PMS surgery [2,4-6]. Some reports state that there was almost no decrease in endothelial cells [4,5], while one study reported a 7.7% decrease over two years [2]. Another study suggested that endothelial cell loss is related to a shorter tube-cornea distance [6]. Additionally, there have been case reports of PMS removal due to corneal endothelial loss five years after surgery [7]. However, no report has documented significant and rapid endothelial cell loss following PMS surgery. We present a rare case of rapid endothelial cell loss after PMS surgery that necessitated tube removal and conversion to trabeculectomy, highlighting a potential complication that has not been widely reported. We believe that this case report will help raise awareness regarding this complication and serve as a reference for its management in similar situations.

## Case presentation

**Figure 1:**
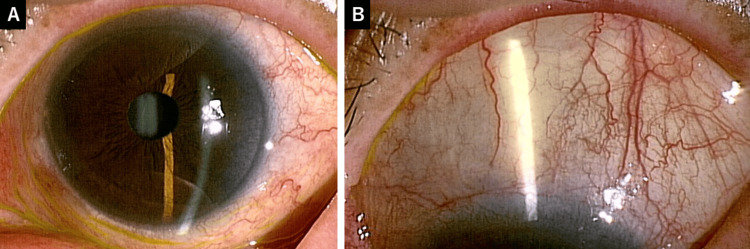
Preoperative findings of the left eye on slit-lamp examination. (A) The anterior segment is normal, with a clear cornea, a deep anterior chamber, and a well-structured iris. (B) The superior conjunctiva is normal, with no signs of scarring.

**Figure 2:**
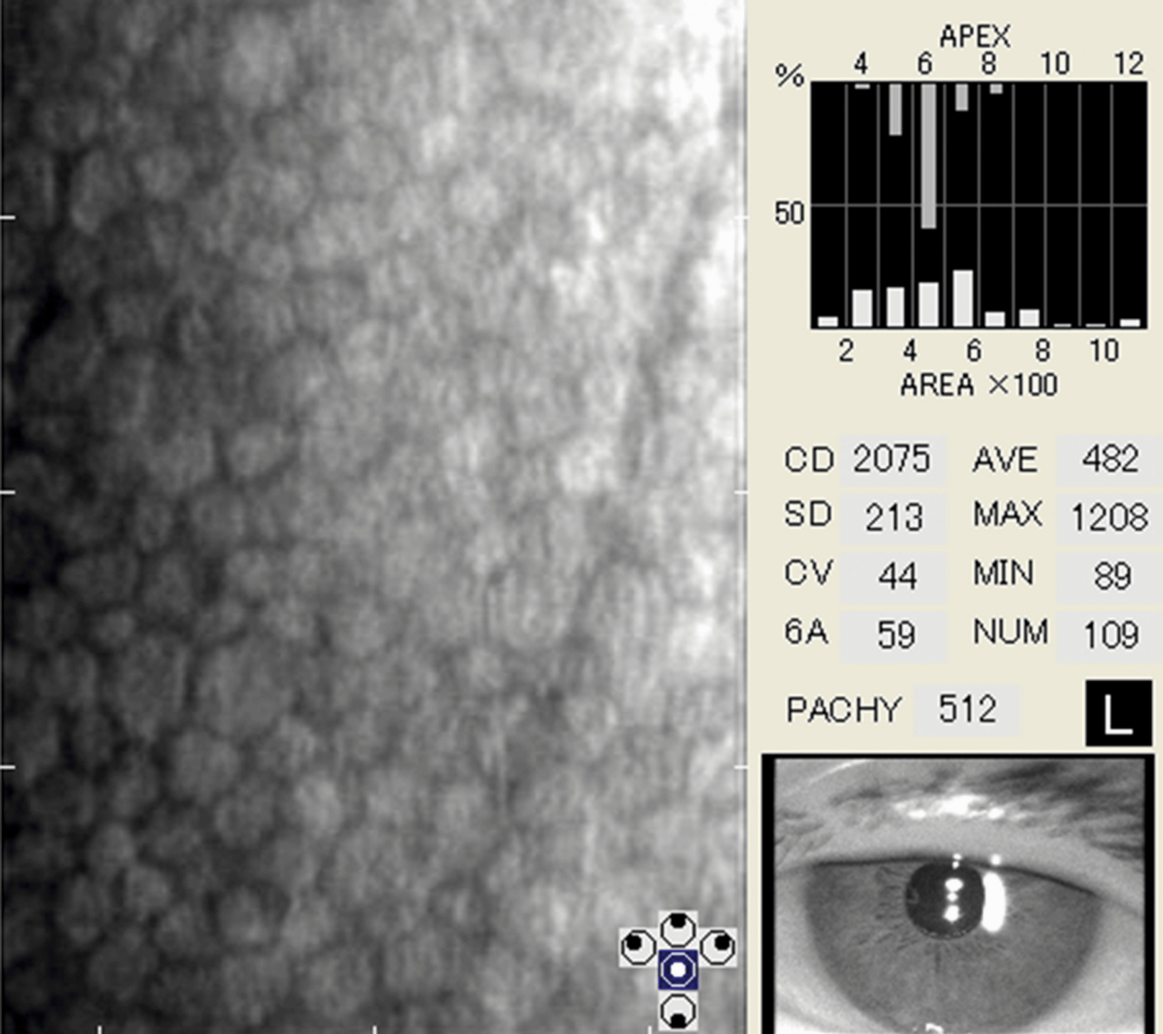
Preoperative specular microscopy findings of the left eye.

An 85-year-old Japanese female presented with advanced bilateral primary open-angle glaucoma (POAG). She had previously undergone cataract extraction and intraocular lens implantation in both eyes. The right eye was already blind due to POAG. Preoperatively, the best-corrected visual acuity was 0.8 in the left eye. Goldmann applanation tonometry showed an IOP of 23 mmHg in the left eye using one glaucoma medication (latanoprost 0.005% + timolol 0.5%). She had allergies to other glaucoma medications (brimonidine tartrate 0.1% and ripasudil hydrochloride hydrate 0.4%), making it difficult to receive additional glaucoma medication. Consequently, the elevated IOP in the left eye could not be controlled with glaucoma medication. The mean deviation in the left eye on the Humphrey visual field 24-2 Swedish Interactive Threshold Algorithm (SITA) standard was -17.18 dB. Slit-lamp and gonioscopic examinations revealed a normal anterior segment of the left eye, particularly in the superior conjunctiva (Figure 1). The anterior chamber depth was 4.8 mm. The preoperative corneal endothelial cell density (CECD) was 2075 cells/mm^2^ (Figure 2). Standalone PMS implantation was scheduled for the left eye.

A corneal suture was placed on the superior cornea to control the eyeball. A 5-mm conjunctival incision was made along the limbus with posterior dissection to create a fornix-based conjunctival flap in the superior temporal quadrant. Mitomycin C (0.4 mg/mL) was applied under Tenon’s capsule for four minutes, followed by irrigation with 100 mL balanced salt solution (BSS). Limited cauterization was performed on episcleral vessels at the PMS insertion site. The sclera was marked 3 mm posterior to the limbus using the provided marking instrument. A scleral tunnel and pocket were created at this site using the provided double-step knife. Subsequently, the PMS was inserted into the anterior chamber through the scleral tunnel with its wings securely inserted into the scleral pocket. However, because the tube tip appeared too close to the cornea after the initial insertion, a second scleral tunnel and a pocket were created. Aqueous flow was confirmed by observing drainage at the posterior PMS tip. The distal portion of the PMS was fixed to the scleral surface using 10-0 Nylon. The conjunctival flap was sutured watertight at the limbus using two wing and mattress sutures with 10-0 Nylon. Postoperatively, a small hyphema was observed. The patient was prescribed postoperative topical medications with 0.5% moxifloxacin and 0.1% betamethasone sodium phosphate, both administered three times daily.

**Figure 3:**
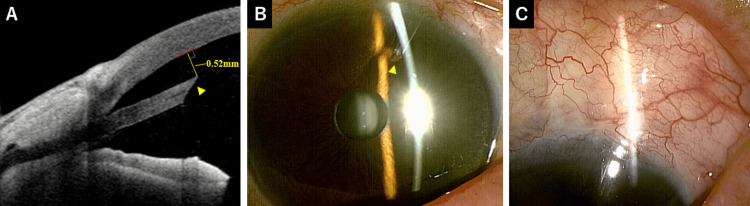
Postoperative findings after PMS surgery. (A) Anterior segment optical coherence tomography image showing the tube tip positioned close to the cornea and inserted bevel down (arrowhead). The tube-cornea distance was 0.52 mm. (B) The tube is visible in the anterior chamber (arrowhead). (C) A diffuse bleb is observed. PMS: PreserFlo MicroShunt

**Figure 4:**
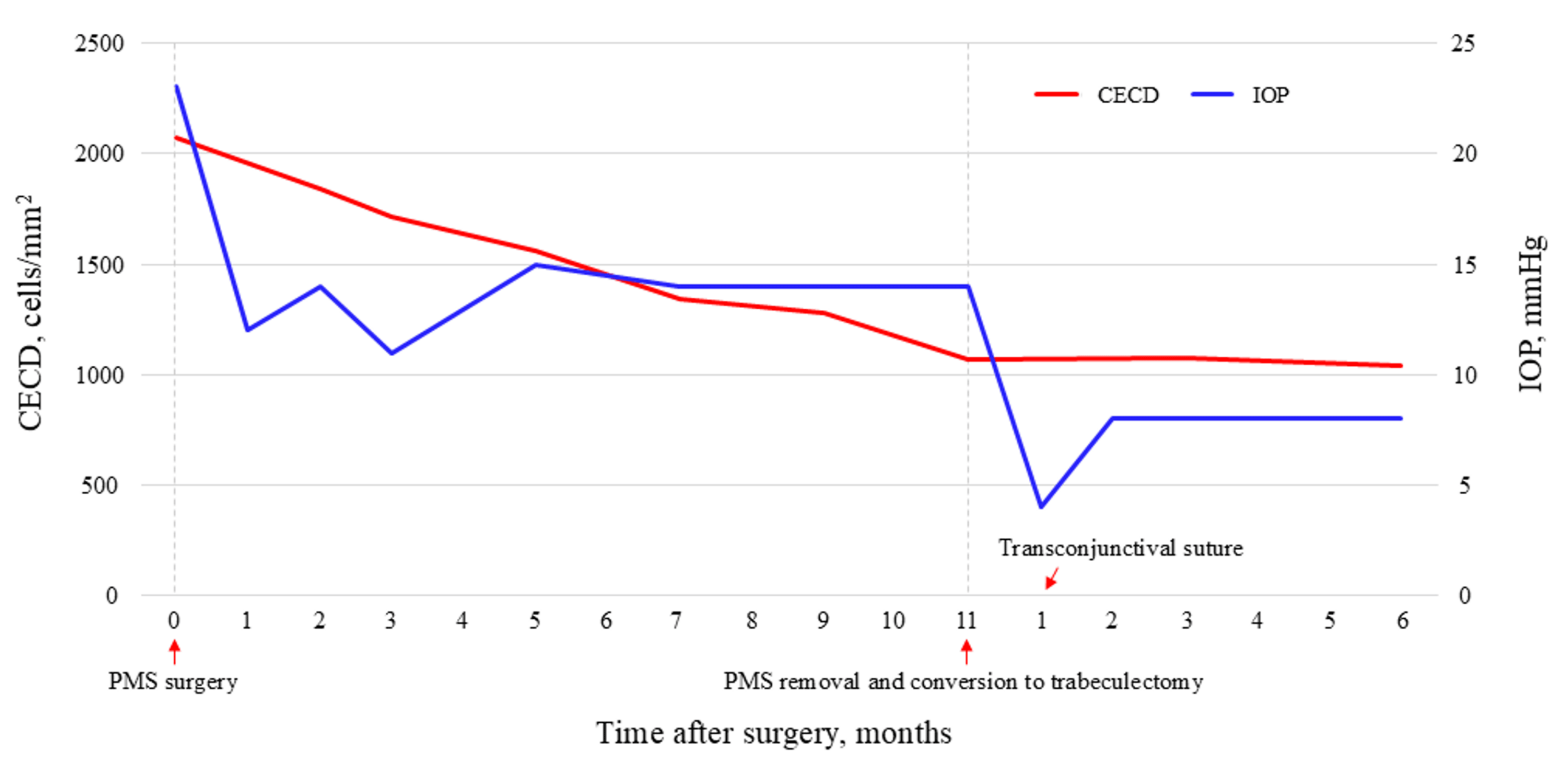
Changes in CECD and IOP over the course of follow-up. CECD: corneal endothelial cell density; IOP: intraocular pressure; PMS: PreserFlo MicroShunt

On postoperative day 1, the IOP in the left eye was 5 mmHg, with a hyphema of 1 mm height and a diffuse bleb. Until two weeks postoperatively, the IOP remained stable at 6-8 mmHg, and the hyphema gradually decreased. The tube tip was positioned close to the cornea and inserted bevel down (Figure 3A). The tube-cornea distance was 0.52 mm. During surgery, when the tube was inserted for the second time, it was placed upside down, resulting in a bevel-down insertion. Gonioscopic examination showed that the tube exited the anterior chamber posterior to the Schwalbe line. No complications related to low IOP, such as choroidal detachment or hypotensive maculopathy, were observed. At one month postoperatively, the IOP was 12 mmHg with a diffuse bleb, and the hyphema was completely resolved (Figures 3B-3C). Thereafter, the IOP remained stable at 14 mmHg without glaucoma medication; however, the CECD rapidly decreased. The CECD decreased to 1842 cells/mm^2^ at two months, 1717 cells/mm^2^ at three months, 1561 cells/mm^2^ at five months, 1339 cells/mm^2^ at seven months, 1282 cells/mm^2^ at nine months, and 1069 cells/mm^2^ (48.5% loss) at 11 months after surgery (Figure 4). Due to the progressive CECD loss, PMS removal was deemed necessary to prevent further deterioration. PMS removal was proposed seven months postoperatively; however, the patient was hesitant and required time to consider the decision. As a result, PMS removal and conversion to trabeculectomy in the same quadrant were scheduled 11 months after surgery.

A corneal suture was placed on the superior cornea to stabilize the eyeball. A conjunctival flap, similar to that used in the PMS surgery, was created in the same superior temporal quadrant. The adhesions around the PMS tip were dissected and the PMS was removed. Limited cauterization was performed on episcleral vessels at the scleral flap site. A 3 mm-wide, half-thickness scleral flap was created. Mitomycin C (0.4 mg/mL) was applied on and beneath the scleral flap as well as under Tenon’s capsule for four minutes, followed by irrigation with 100 mL BSS. A deep limbal block excision and peripheral iridectomy were performed to create a fistula in the anterior chamber. The scleral flap was secured using two sutures of 10-0 Nylon. The conjunctival flap was sutured watertight at the limbus with two wing and mattress sutures using 10-0 Nylon. The patient received postoperative topical medications with 0.5% moxifloxacin and 0.1% betamethasone sodium phosphate three times daily.

One day after the secondary surgery, the IOP was 7 mmHg, and the patient had a diffuse bleb. Two weeks after the secondary surgery, the IOP decreased to 3 mmHg without any complications related to low IOP. One month after the secondary surgery, IOP was 4 mmHg, and the patient developed severe choroidal detachment and hypotensive maculopathy. Owing to excessive filtration, three transconjunctival sutures using 10-0 Nylon were placed in the scleral flap. One week after transconjunctival suturing, the IOP increased to 10 mmHg, and both choroidal detachment and hypotensive maculopathy resolved. The IOP remained stable thereafter, measuring 8 mmHg six months after the secondary surgery (Figure 4). The CECD was 1075 cells/mm^2^ at three months and 1038 cells/mm^2^ at six months after the secondary surgery (Figure 4). The decline in CECD ceased following secondary surgery. The best-corrected visual acuity was 0.7 in the left eye at six months after the secondary surgery.

## Discussion

This case highlights the rare occurrence of rapid corneal endothelial cell loss following PMS surgery, ultimately necessitating PMS removal and conversion to trabeculectomy. While PMS has been reported to have a favorable safety profile with fewer postoperative interventions and a lower incidence of hypotony than trabeculectomy, endothelial cell loss can occur, particularly when the tube is positioned close to the cornea [2,6]. However, previous reports have not documented significant or rapid endothelial cell loss after PMS surgery. In this case, despite stable IOP control without glaucoma medications, the patient's CECD declined rapidly over 11 months, from 2075 cells/mm² preoperatively to 1069 cells/mm². This progressive endothelial cell loss prompted the decision to remove PMS and perform trabeculectomy in the same quadrant. Notably, after PMS removal, the decline in CECD ceased, suggesting a direct relationship between PMS placement and endothelial cell loss in this patient.

The exact mechanism underlying accelerated endothelial cell loss remains unclear. One plausible explanation is the proximity of the PMS tube tip to the cornea, which could have caused mechanical trauma or chronic low-grade inflammation leading to progressive endothelial damage. The PMS tube should be inserted with the bevel up; however, in this case, it was inserted with the bevel down, which may have caused the tip of the tube to be positioned even closer to the cornea. Additionally, the presence of an aqueous outflow pathway near the endothelium may have contributed to localized turbulence or chemical stress, further exacerbating endothelial cell loss. In fact, even in tube shunt surgeries, such as those performed with the Baerveldt glaucoma implant (Abbott Medical Optics, Abbott Park, IL, USA) and Ahmed glaucoma valve (New World Medical, CA, USA) implants, endothelial cell loss is accelerated when the tube is in close proximity to the cornea [8-10]. In our previous report on endothelial cell loss following Baerveldt glaucoma implant surgery, the average tube-cornea distance was 1.7 mm [9]. In contrast, the tube-cornea distance in the present case was only 0.52 mm, indicating a significantly closer proximity to the cornea. However, a previous study examining the relationship between tube-cornea distance and endothelial cell loss after PMS surgery reported that when the distance exceeds 0.5 mm, CECD decreases by only 1% at one year postoperatively [6]. Therefore, the 0.52 mm distance in the present case might have been sufficient to prevent significant endothelial cell loss. Furthermore, the bevel-down orientation of the tube may have altered the aqueous humor flow compared with the bevel-up position, potentially leading to endothelial trophic disturbances. Among other possible explanations, endothelial cell loss may be induced by the patient-specific endothelial vulnerability due to age, previous surgery or glaucoma medications, trophic disturbances resulting from impaired aqueous humor flow, or the cytotoxic effects of intraoperative Mitomycin C. Although it is difficult to determine the extent to which these factors contributed, their impact is considered minimal, as the endothelial cell loss ceased following the removal of the PMS.

After PMS removal and trabeculectomy, the patient initially experienced excessive filtration, which resulted in hypotensive maculopathy and severe choroidal detachment. These complications were effectively managed with transconjunctival sutures, stabilizing the IOP to 8 mmHg by six months postoperatively. Importantly, the rapid decline in CECD was halted following PMS removal, reinforcing the hypothesis that PMS placement was the primary factor contributing to endothelial cell loss.

## Conclusions

This case underscores the importance of careful tube positioning during PMS surgery to minimize endothelial damage. Clinicians should closely monitor corneal endothelial health following PMS implantation, particularly in cases where the tube is positioned near the cornea. If progressive endothelial cell loss is detected despite stable IOP, timely intervention, including PMS removal, may be necessary to prevent irreversible corneal decompensation. Further studies are needed to better understand the long-term effects of PMS on corneal endothelial health and to establish optimal surgical techniques to minimize this risk.
